# HP1BP3 is a novel histone H1 related protein with essential roles in viability and growth

**DOI:** 10.1093/nar/gkv089

**Published:** 2015-02-08

**Authors:** Benjamin P. Garfinkel, Naomi Melamed-Book, Eli Anuka, Michael Bustin, Joseph Orly

**Affiliations:** 1 Department of Biological Chemistry, The Alexander Silberman Institute of Life Sciences, The Hebrew University of Jerusalem, Jerusalem 91904, Israel; 2 Bio-Imaging Unit, The Alexander Silberman Institute of Life Sciences, The Hebrew University of Jerusalem, Jerusalem 91904, Israel; 3 Protein Section, Laboratory of Metabolism, Center for Cancer Research, NCI, National Institutes of Health, Bethesda, MD 20892, USA

## Abstract

The dynamic architecture of chromatin is vital for proper cellular function, and is maintained by the concerted action of numerous nuclear proteins, including that of the linker histone H1 variants, the most abundant family of nucleosome-binding proteins. Here we show that the nuclear protein HP1BP3 is widely expressed in most vertebrate tissues and is evolutionarily and structurally related to the H1 family. HP1BP3 contains three globular domains and a highly positively charged C-terminal domain, resembling similar domains in H1. Fluorescence recovery after photobleaching (FRAP) studies indicate that like H1, binding of HP1BP3 to chromatin depends on both its C and N terminal regions and is affected by the cell cycle and post translational modifications. HP1BP3 contains functional motifs not found in H1 histones, including an acidic stretch and a consensus HP1-binding motif. Transcriptional profiling of HeLa cells lacking HP1BP3 showed altered expression of 383 genes, suggesting a role for HP1BP3 in modulation of gene expression. Significantly, *Hp1bp3^−/−^* mice present a dramatic phenotype with 60% of pups dying within 24 h of birth and the surviving animals exhibiting a lifelong 20% growth retardation. We suggest that HP1BP3 is a ubiquitous histone H1 like nuclear protein with distinct and non-redundant functions necessary for survival and growth.

## INTRODUCTION

Organization of the vast genomes of eukaryotic cells in the confines of the nucleus, while still allowing tightly regulated access to transcription factors demands a complex system of dynamic compaction. This is achieved by the packaging of DNA into chromatin. The building block of chromatin is the nucleosomal core particle containing a histone octamer around which 147 bp of DNA are wrapped (1). The dynamic nature of the chromatin fiber is mediated by a network of numerous nuclear proteins that bind to and remodel nucleosomes, allowing the constant modulation of local chromatin structure (2–5). The network of binding proteins includes many structural chromatin binding proteins, among them the histone H1 gene family and the high mobility group (HMG) proteins.

The present study describes a novel chromatin binding protein, heterochromatin protein 1 binding protein 3 (HP1BP3, HP1-BP74), originally discovered as a binding protein of the heterochromatin protein HP1 (6). The findings described herein suggest that HP1BP3 is related to the linker histone H1 family. Histone H1 is a family of lysine-rich proteins that confer higher-order organization to chromatin by binding to the surface of nucleosomes and interacting with nucleosomal DNA at the entry and exit points (7). The H1 gene family is the fastest evolving of the histone families and through processes of gene duplication, mutation and selection, has grown from one H1 gene in single cell eukaryotes to no less than 11 different mammalian H1 subtypes (7,8). The subtypes differ from each other in a variety of aspects, including chromatin dynamics (9–12), cell type and tissue-specificity (13–16), developmental regulation (17,18), evolutionary stability (19) and posttranslational modifications (9–11,20). Furthermore, global gene expression analyses in various cell types have revealed that the histone H1 variants control the expression of different subsets of genes (12,21). Surprisingly, in spite of all of these differences, knockout of single somatic H1 subtypes in mice does not lead to any obvious phenotype (22–24).

In an attempt to the reveal the physiological relevance of HP1BP3, we characterized its tissue distribution in a murine model and studied the major determinants controlling the association of HP1BP3 with chromatin. We also explored the impact of HP1BP3 knockdown on transcriptional profiling in HeLa cells and in a genetically engineered mouse model lacking expression of this protein. We find that HP1BP3 is a novel histone H1 related protein endowed with unique chromatin binding determinants and involved in the modulation of gene expression. Surprisingly, unlike individual members of the histone H1 gene family, the murine HP1BP3 plays vital and non-redundant roles in viability and growth.

## MATERIALS AND METHODS

### Animals

*Hp1bp3^tm1a(EUCOMM)Wtsi^* mice were acquired from the European Conditional Mouse Mutagenesis Program (EUCOMM). In these mice, a FlipROSAβGeo cassette (25) was inserted into intron 7 of the *Hp1bp3* gene, leading to the production of a truncated transcript. Genotypes were determined at weaning using polymerase chain reaction (PCR). The mice were maintained under a schedule of 12 h light, 12 h dark with food and water *ad libitum*, and were treated in accordance with the National Institutes of Health Guide for the Care and Use of Laboratory Animals. All protocols had the approval of the Institutional Committee on Animal Care and Use, The Alexander Silverman Institute of Life Sciences, The Hebrew University of Jerusalem. Mice were weaned at the age of 21 days and were weighed bi-weekly from then on. For newborn blood analysis, pups were killed by decapitation, blood was pooled and serum was separated.

### Phylogenetic analysis

Molecular evolutionary analysis was conducted using MEGA version 5.03 software (26,27). Amino acid sequences of the globular domains of human and chicken H1 and HP1BP3 were retrieved from the NCBI protein sequence database. Alignments were done using ClustalW and the amino acid sequence divergence was estimated by means of the uncorrected differences (p-distance). The phylogenetic tree was reconstructed using the neighbor-joining (NJ) tree-building method (28). The reliability of the resulting topologies was tested by the bootstrap method (1000 replicates). We rooted the tree using the *H1* gene of the mycetozoan *Dictyostelium discoideum*, representing one of the most primitive eukaryotes for which an H1-related protein with a globular domain has been characterized (29).

### Expression constructs and cell culture

All primers used for cloning of HP1BP3 and its mutants are described in Supplementary Table S1. cDNA was prepared from C57BL/6 mouse embryos and *Hp1bp3* was cloned into pcDNA3.1+ (Life Technologies) using KpnI and EcoRV. The expression constructs GFP-HP1BP3^wt^, GFP-HP1BP3^ΔCTD^, GFP-HP1BP3^ΔNTD^, GFP-HP1BP3^ΔDE+CTD^ and additional deletion mutants were subcloned into pEGFP-C1 and pmCherry-C1 using BspEI and Sal1. Fynnzyme's site directed mutagenesis protocol was used for GFP-HP1BP3^ΔDE^ and the Quickchange method was used for GFP-HP1BP3^V257E^. GFP-H1.2, GFP-HP1α, GFP-HP1β and GFP-HP1γ were all kindly provided by Eran Meshorer and Tom Misteli (30,31). mHMGN5-YFP has been described before (32) and the PCNA-RFP expression plasmid was a kind gift from Dr M. Cristina Cardoso and has been described before (33). HeLa and NIH-3T3 cells were cultured in Dulbecco's modified Eagle's medium (DMEM) supplemented with 10% fetal bovine serum (FBS) and for stable cells 200 μg/ml G418. For live cell experiments, cells were seeded in μ-slides (Ibidi GmbH), using either pools of stably expressing cells or transiently transfected cells.

For isolation of mouse embryonic fibroblasts (MEF), uteri from 13.5-day-pregnant mice were washed with phosphate-buffered saline (PBS). The head and visceral tissues were removed from isolated embryos. The remaining bodies were washed in fresh PBS, minced, transferred into a 0.1 mM trypsin/1 mM ethylenediaminetetraacetic acid (EDTA) solution (3 ml per embryo), and incubated at 37°C for 30 min. After trypsinization, an equal amount of medium (3 ml per embryo DMEM containing 10% FBS) was added and pipetted up and down a few times to help with tissue dissociation. Cells were collected by centrifugation (200 × *g* for 5 min at 4°C) and resuspended in fresh medium. The cells were cultured in DMEM supplemented with 10% FBS at 37°C with 5% CO_2_.

For stable expression of GFP-HP1BP3^wt^, GFP-HP1BP3b^wt^, GFP-HP1BP3^ΔCTD^, GFP-HP1BP3^ΔNTD^, HeLa cells were transfected in 6-well tissue culture plates using lipofectamine and 3 days later transferred to 14 cm plates and grown for 2 weeks in medium containing 500 µg/ml G418. Cells were then collected and sorted using a FACSAria Cell-Sorting System (Becton Dickinson). Cells were grown in selection medium for another week, then sorted a second time. For transient transfections, cells were grown up to 30–40% confluence and transfected with Lipofectamine (Life Technologies) according to the manufacturer's instructions. Cells were then incubated overnight before performing Fluorescence recovery after photobleaching (FRAP) experiments. Where indicated, 100 ng/ml of Trichostatin A (TSA) (Sigma-Aldrich) or 1 ug/ml Staurosporine (Sigma-Aldrich) were added directly to the wells 2 and 1.5 h respectively before beginning FRAP measurements.

### Salt extraction

HeLa cells were washed with 1× PBS and then in washing buffer (20 mM HEPES [pH 7.0], 10 mM KCl, 10 mM MgCl2, 20% glycerol, 0.1% Triton X-100, 0.5 mM DL-Dithiothreitol (DTT), 1 mM orthovanadate, complete protease inhibitor, 0.25 mM) Phenylmethanesulfonyl fluoride (PMSF). Finally, cells were resuspended in 200 μl of the washing buffer and NaCl was added to final concentration of 0, 100, 200, 300, 400 and 500 mM. Extraction was performed on ice for 15 min, with over-the-top mixing every 2–3 min. Cells were centrifuged at 15 000 *g* for 5 min. The pellet and the supernatant fraction were collected. Laemmli buffer supplemented with protease inhibitors was added to both fractions, followed by brief sonication and boiling.

### Fluorescence recovery after photobleaching and live cell imaging

FRAP experiments were performed using a FV-1000 confocal microscope (Olympus, Japan) with a CO_2_/temp incubator (LIS, Basel, Switzerland). The microscope is equipped with a sim scanner which enables us to bleach using the 405 nm laser line while scanning with the 488 nm laser line. All experiments were done using a 60x/1.35 oil objective. We performed FRAP experiments by exposing a region of ∼5 μm in cell nuclei to 100% laser intensity for 0.45 ms. Imaging was typically performed at 1% laser intensity. The interval between image scans varied depending on the duration of recovery in an initial pilot experiment and was either 1 s or 250 ms for slow and fast recovery respectively. Recovery was considered complete when the intensity of the photo-bleached region stabilized (that is, the curve flattened). Typically 10 pre-bleach and 180 post-bleach frames were recorded for each series. As cells tend to move during the imaging time, we corrected using ImageJ StackReg plugin prior to quantitative analysis. The mean fluorescence intensities of the bleached region for each time point were normalized to the mean of the last five pre-bleach values. These values were divided by the respective total nuclear fluorescence in order to correct for total loss of nuclear fluorescence. While the expression level did not have any apparent effect on recovery dynamics, we used only cells expressing low amounts of the Green Fluorescent Protein (GFP) fusion protein. For each construct and condition 6–20 nuclei were averaged and the mean curve as well as the standard error of the mean (SEM) was calculated. Half times of recovery were calculated from the mean curves.

For determination of cell cycle phase, RFP-PCNA was observed prior to beginning of FRAP. For nuclear localization signal (NLS) analysis, cells were treated with 0.25 μg/ml Hoechst 33342 20 min prior to imaging.

To assess the enrichment of the GFP-HP1BP3^WT^ and GFP-HP1BP3^V257E^ constructs in pericentromeric heterochromatin, MEF cells were transfected with the constructs, and live cells were treated with 0.25 μg/ml Hoechst 33342 for DNA visualization. Confocal optical sections were collected from 20 cells and Image analysis was performed with ImageJ. To measure protein enrichment at pericentromeric heterochromatin, a single random pericentromeric focus and three nucleoplasmic areas of blue fluorescent Hoechst staining were circled using the freehand lasso tool. The histogram function was then used to measure the mean intensity for each area in the green channel, giving a value for GFP intensity. Protein enrichment was calculated by dividing the mean GFP intensity at pericentromeric heterochromatin by the mean GFP nucleoplasmic intensity. While the expression level did not have any apparent effect on enrichment, we used only cells expressing low amounts of the GFP fusion proteins.

### Antibodies

The anti-HP1BP3 antibody was created by injecting guinea pigs with the peptide STRETPPKSKLAEGEEEKPEPD-C corresponding to amino acids 47–68 of the murine protein. Peptide synthesis, keyhole limpet hemocyanin (KLH) conjugation and immunization of guinea pigs were carried out by Peptide Specialty Laboratories GmbH, Heidelberg, Germany. Anti-Histone H3 (ab1791) and anti-HP1α (ab77256) antibodies were from Abcam. Anti H3K9me3 antibody was from Cell signaling (#9754) and anti-Tubulin was purchased from Sigma-Aldrich (T5168).

### RT-PCR analysis

Total RNA was extracted and isolated with 1 ml TRI reagent (Sigma-Aldrich) according to the manufacturer's instructions. First strand cDNA synthesis was performed on 1 μg total RNA with RevertAid M-MuLV Reverse Transcriptase (Thermo Scientific) according to the manufacturer's instructions. PCR was performed on 75 ng cDNA with Phusion polymerase (Thermo Scientific) and the PCR products were analyzed by electrophoresis on 1% polyacrylamide gel in Tris-Acetate-EDTA buffer.

### Tissue lysis and SDS-PAGE

Eight week old male and female C57BL/6N mice were sacrificed by CO_2_ followed by cervical dislocation. Tissues were extracted, washed twice in PBS, diced and 100 mg were homogenized in 0.5 ml RIPA buffer in dounce homogenizers. Extracts were then left on ice for 30 min, followed by 10 min of centrifugation at 10 000 × *g*. The supernatant was separated from pellet and fat, and centrifuged again. This was repeated until the sup was cleared, at which point it was resuspended in sample loading buffer. Total protein in each sample was quantified using the bradford assay. Fifteen micrograms protein from each sample were separated by electrophoresis in 7 or 12% polyacrilamide gels and transferred onto a polyvinylidene difluoride membrane (Immobilon P; Millipore, Billerica, MA, USA) by semidry transfer. After transfer, the membranes were blocked with 1% milk in PBS containing 0.1% Tween-20 (PBST) for 30 min at room temperature and incubated for 1 h at room temperature with anti-HP1BP3 antibodies and anti Histone H3 antibodies. The membrane was washed with PBST, and was then incubated with goat anti-guinea pig IgG secondary antibody coupled to peroxidase (Jackson immunoresearch) and observed using enhanced chemical luminescence (Cell signaling).

### Tissue fixation, immunohistochemistry and immunocytochemistry

For immunofluorescent labeling of cell culture, cells were fixed with 4% paraformaldehyde (PFA) for 10 min and permeabilized with 1% Triton X-100 for 4 min prior to labeling. For mouse tissues, mice were killed by cervical dislocation and tissues were surgically removed and placed for 24 h in fresh cold 4% para-formaldehyde in PBS. Samples were then embedded in paraffin and 4 μm sections were placed on slides. After rehydration, slides were placed in a microwave oven for 15 min in TE buffer pH 9.0 for antigen retrieval. Immunofluorescent staining was performed for tissues and cell cultures as previously described (34), using a 1:100 dilution of antibodies. Confocal laser scanning microscopy was performed using an FV-1000 confocal work station (Olympus, Japan), based on an IX81 inverted microscope; objectives used were 10X/0.3, 20X/0.75, 40X/1.3 oil and 60X/1.35 oil.

### Cell permeabilization and exposure to micrococcal nuclease

Chromosomal DNA from HeLa and MEF cells was analyzed according to the procedure described by Zaret (21) with modifications. MEFs from *Hp1bp3^+/+^* or *Hp1bp3^−/−^* were grown to confluency on 14 cm plates. HeLa cells were transfected with either HP1BP3 specific or negative control siRNA 72 h prior to harvest. Cells were trypsinized, then washed twice with PBS and twice with reticulocyte standard buffer (RSB); 10 mM Tris pH 7.4, 10 mM NaCl, 3 mM MgCl_2_), then divided into samples of 1.5 × 10^6^ cells. Permeabilization was with 0.1 mg/ml lysolecithin (Sigma Aldrich) in 200 μl RSB for 1 min, followed by immediate dilution with 1 ml RSB and centrifugation. Cells were resuspended in 200 μl RSB containing 3 mM CaCl_2_ and incubated with 0.05 units of micrococcal nuclease (Sigma Aldrich) at 37°C. The digestion was stopped by the addition of an equal volume of stop solution (20 mM Tris pH 7.4, 200 mM NaCl, 2 mM EDTA, 2% sodium dodecyl sulphate, 3 units Proteinase K) at indicated time points. After overnight incubation at 37°C, DNA was purified using a standard phenol–chloroform extraction, followed by treatment with 10 μg RNase A (Sigma Aldrich) and ethanol precipitation. Genomic DNA was analyzed on a 1.5% agarose gel in Tris-Acetate EDTA (TAE) (40 mM Tris-acetate, pH 8.3, 1 mM EDTA) buffer containing ethidium bromide.

### Microarray hybridization, analysis and real-time RT-PCR

Total RNA was isolated from triplicates of HeLa cells 72 h after transfection with HP1BP3 specific or negative control siRNA. Samples were quantified by spectroscopy and purity was analyzed by the 260:280 absorbance ratio. RNA quality and integrity were assessed using Bioanalyzer 2100 (Agilent Technologies, Santa Clara, CA, USA) with all samples having high-quality RNA [RNA integrity number (RIN) = 9.7–10]. Hybridization was performed with Human Gene 1.0 ST arrays (Affymetrix, Inc, Santa Clara, CA, USA). Briefly, 100 ng of total RNA from each sample were reverse transcribed to cDNA, followed by overnight *in* *vitro* transcription to generate cRNA, which was reverse transcribed and the 5.5 μg of sense cDNA were fragmented and labeled. The quality of cDNA and fragmented cDNA was assessed in the Agilent bioanalyzer. Microarrays were hybridized, washed, stained and scanned according to the protocol described in the WT sense target labeling assay manual from Affymetrix (version 4; FS450_0007).

Raw expression values from the Affymetrix mouse gene 1.0 ST chip were analyzed and normalized using Partek Genomics Suite 6.4 (Partek Incorporated). *P*-values acquired by ANOVA analysis were corrected with False Discovery Rate (FDR) for *q*-values (35).

For quantitative qRT-PCR analysis, 2 μg of RNA were converted to cDNA using the RevertAid M-MuLV Reverse Transcriptase (Thermo Scientific) and the real-time RT-PCR reaction was carried out using the KAPA SYBR Fast kit (KAPA Biosystems) for 40 cycles. Primers are listed in Supplementary Table S1.

## RESULTS

### HP1BP3 is a member of the H1 linker histone multigene family

**Figure 1.**
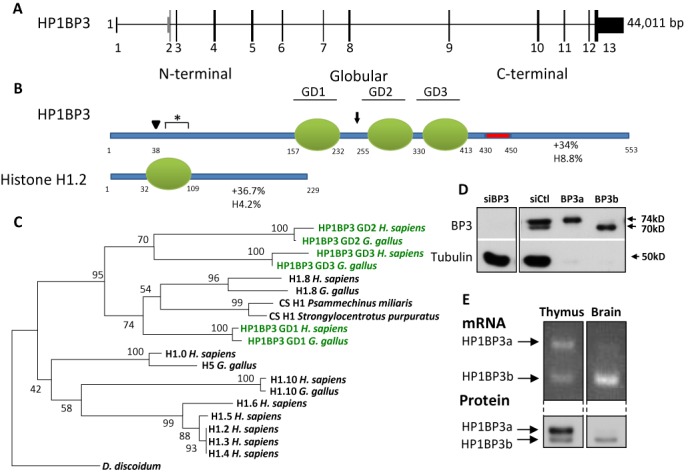
HP1BP3 is structurally and evolutionarily related to the histone H1 family. (**A**) The genomic locus of HP1BP3 on human chromosome 1p36, with 13 exons. Introns are represented by a flat line, UTRs by narrow bars and coding exons by wide bars. The coding sequence begins in exon 2 (Gray), which is sometimes skipped by alternative splicing, shifting the translation start to exon 3. (**B**) A schematic representation of HP1BP3 shows that it can be divided into three general regions. The N terminal region (NTD) spans the first 157 amino acids and is very rich in Glu. The middle of the protein is comprised of three consecutive domains with high sequence similarity to the H1 globular domain (GD). Finally, a poly-aspartate tract (DE) precedes the very basic C terminal region (CTD). This tripartite organization resembles that of all known vertebrate histone H1 subtypes, represented here by H1.2. The percentage of positively charged and hydrophobic residues in the CTDs is shown beneath the CTD regions. The residue numbers at start and end points of the different regions are listed. HP1BP3 also contains a consensus HP1 binding motif (black arrow). Arrowhead marks the beginning of translation of the alternatively spliced variant. Asterisk marks the epitope used for generation of polyclonal antibody. (**C**) Phylogenetic analysis of HP1BP3 within the H1 gene family. Globular domains from human and chicken H1 proteins were analyzed using uncorrected p-distances and a phylogenetic tree was constructed using the Neighbor-Joining method. The numbers for interior branches represent bootstrap values based on 1000 replications. The GDs of HP1BP3 are highlighted in green for clarity. (**D**) Two splice variants of HP1BP3 are expressed and translated in HeLa cells. Cells were transfected with either control or specific siRNA, or with expression vectors for HP1BP3a or HP1BP3b and analyzed with anti HP1BP3 antibody. Due to the high expression of the ectopic protein, 10-fold less extract was loaded, and hence no tubulin is apparent in lanes 3 and 4. Note also that an irrelevant gap was excised from the gel. (**E**) The two splice variants are observable in mouse tissues and are tissue specific. RNA and protein were extracted from mouse tissues and analyzed by RT-PCR with splice specific primers and western blot with anti HP1BP3 antibody. Note that for brevity, the thymus and brain were selected out of the full analysis gel as representative tissues. For the complete set of tissue transcript splice patterns, see Supplementary Figure S1.

BLAST analysis of HP1BP3 showed that it is present in all vertebrates. In humans, the *HP1BP3* gene is located on chromosome 1p36, in the center of a region syntenic to the mid-distal mouse chromosome 4 and is composed of 13 exons (Figure 1A). The protein product of the *HP1BP3* gene is 553 amino acids long and contains three regions with high sequence homology to the globular domain of members of the histone H1 gene family (Supplementary Figure S1A). In addition to the globular domains, two more regions can be clearly discerned in the primary sequence of HP1BP3 (Figure 1B). A glutamate rich N-terminal domain (NTD) of 100 amino acids is predicted to be unstructured and followed by the three globular domains, termed GD1, GD2 and GD3. Downstream of GD3, a 20 amino acid poly aspartate/glutamate tract (DE) is followed by a highly basic and unstructured 100 amino acid long C-terminal domain (CTD). While the sequence of the CTD is not similar to that of any H1 subtype, the amino acid composition is strikingly close to that of the CTDs of the gonadal subtypes H1.8 (formerly H1foo; see (36) for new nomenclature) and H1.6 (not shown). This layout of globular domains flanked by an unstructured NTD and a very basic CTD is highly reminiscent of the tri-partite linker histone structure (Figure 1B). HP1BP3 is highly conserved in mammals, with 89% identity and 93% similarity between the human and murine primary sequences. The conservation is particularly striking in the globular domains where the human and murine sequences show 98% identity.

In order to place HP1BP3 within its evolutionary context, we constructed a phylogenetic tree of the vertebrate H1 gene family using the conserved globular domains of the different subtypes, as the NTD and CTD regions are too divergent (Figure 1C). Our analysis places all three globular domains of HP1BP3 within the H1 gene family. GD1 is on the same branch as histone H1.8 and the related mollusk cleavage stage H1, suggesting that a duplication of H1.8 led to the creation of HP1BP3. GD2 and GD3 are more distant, their closest neighbors being themselves and GD1, indicating that two further internal duplications of GD1 led to the current sequence, with three consecutive globular domains. These results are supported by previous analyses of the known H1 subtypes (8,37), which described the sequential divergence of the oocyte specific H1.8, followed by H1.0, H1.10, H1.6 and finally the somatic subtypes H1.1–H1.5.

### HP1BP3 is widely and differentially present in the nuclei of mouse tissues and cells

**Figure 2.**
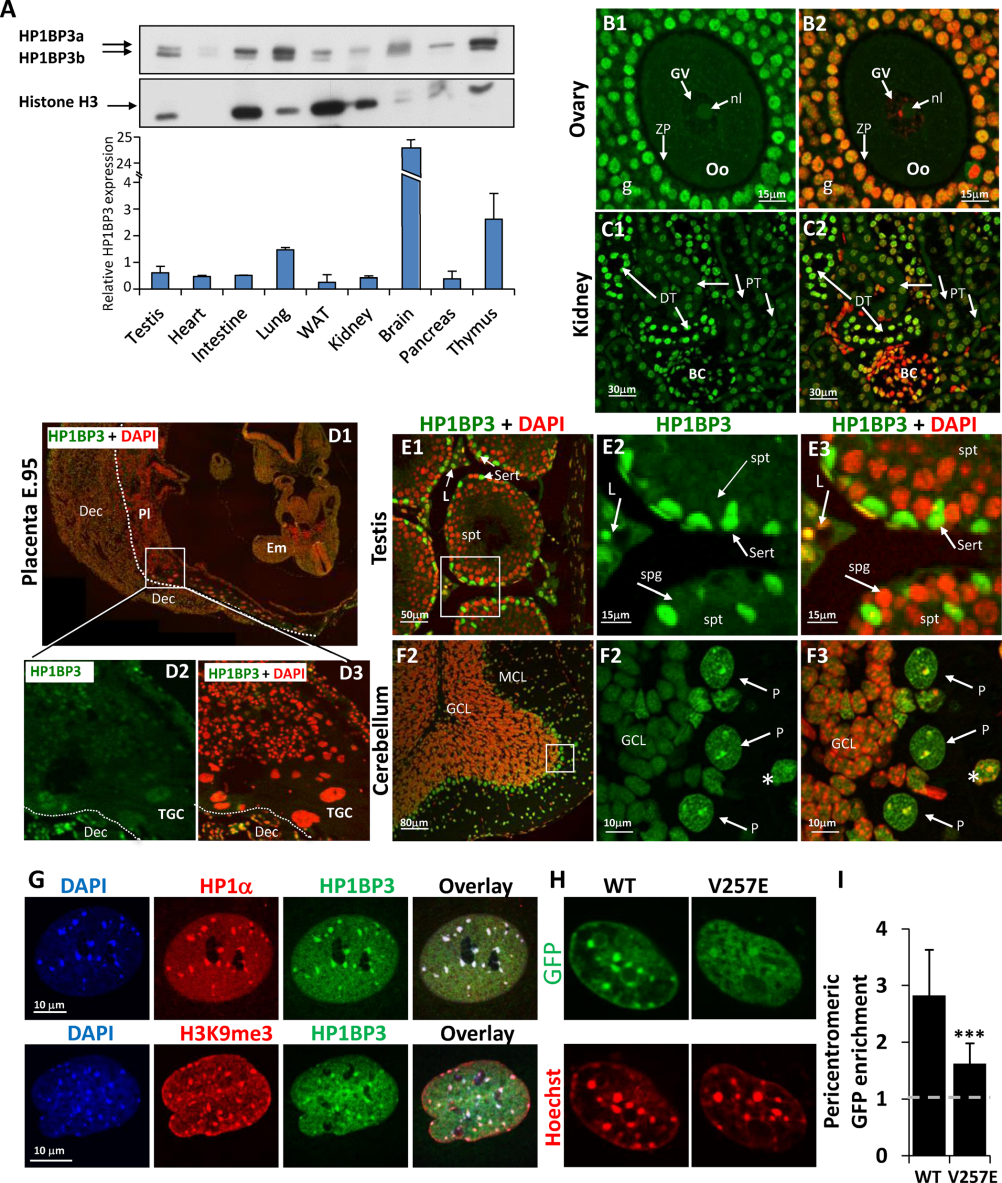
Expression and localization of HP1BP3. (**A**) HP1BP3 is ubiquitously expressed in mouse tissues. HP1BP3 levels in whole cell extracts from a range of moue tissues were analyzed by western blot. Since HP1BP3 is a nuclear protein, we divided its levels by those of histone H3 to achieve average ‘per cell’ levels (bar graph). (**B**–**F**) Immunohistochemical analysis of HP1BP3 expression within tissues. Mouse tissues (4 μm paraffin sections) were prepared for confocal microscopy (78) and stained with anti-HP3BP3 (green) and DAPI (DNA, artificial red). (B1 and B2) Staining of ovary shows high expression in the granulosa (g) and corona radiata (CR) cells, but no expression in the Oocyte (Oo). ZP, zona pellucida; GV, germinal vesicle; nl, nucleolus. (C1 and C2) Staining of kidney shows cell type specific variance in expression levels, with high nuclear content in cells of the distal tubules (DT) and lower in the proximal tubules (PT). BC, Bauman capsule. (D1–D3) A whole implantation site from embryonic day 10.5 was sectioned in paraffin and stained immunohistochemically for HP1BP3 expression. Em, embryo; Pl, placenta; Dec, decidua; TGC, trophoblast giant cells. (E1–E3) Staining of testis where HP1BP3 is highly expressed in Sertoli (Sert) and Leydig (L) cells and completely absent from spermatogonia (spg) and spermatids (spt). (F1–F3) Staining of the cerebellum shows high levels of HP1BP3 in the Purkinje cells (P arrows) and the molecular cell layer (MCL and *), and to a lower extent in the granular cell layer (GCL). (**G**) MEF cells were fixed and labeled with DAPI (blue), HP1BP3 (green) and either HP1α or H3K9me3 (red). Dense pericentromeric foci can be observed in all cases. (**H**) Live MEFs expressing either GFP-HP1BP3^WT^ or GFP-HP1BP3^V257E^ were treated with Hoechst to observe DNA. (**I**) Quantification of HP1BP3 localization. Bars represent the ratio of fluorescence intensity at enriched foci relative to background. Absence of localization results in a homogenous distribution within the nucleus, corresponding to a ratio of 1 (dashed gray line). High enrichment of GFP-HP1BP3^WT^ was observed in pericentromeric foci and this enrichment was lost in GFP-HP1BP3^V257E^ (*P* < 0.001). Data are presented as mean enrichment in 20 nuclei from each group, ± SD.

In order to define the tissue and cell expression patterns of HP1BP3 we generated antiserum to amino acids 47–68 of the human protein (Figure 1B). The antibody is highly specific and siRNA for HP1BP3 completely abolishes the signal in HeLa cells (Figure 1D). Interestingly, the specific signal includes two bands at 74 and 70 kD, which could suggest a splice variant. A search of the Refseq database did indeed show cases of exon 2 being skipped, which would lead to a product predicted to lack the first 38 N-terminal amino acids (Figure 1B, arrowhead). Western blot of HeLa cells expressing either the complete or the exon skipped coding sequence confirmed the presence of the two variants, with the ectopic bands corresponding to the two endogenous ones that we named HP1BP3a and HP1BP3b. Both the mRNA and protein products of these splice variants were observed in a large number of human and mouse cell lines (not shown). The splice variants were also observed in mouse tissues, where they are differentially expressed (Figure 1E and Supplementary Figure S1B). Since HP1BP3a appears to be the major variant in most tissues, we will refer to it as the canonical variant throughout the rest of this work. At least one variant of HP1BP3 was seen in all tissues examined. To assess the relative amounts of total cellular HP1BP3 in tissues, protein levels were normalized to those of histone H3 (Figure 2A). We found similar levels in all tissues but the brain, which has 10-fold higher expression than any other tissue.

While expression of HP1BP3 was ubiquitous at the whole tissue level, analysis of immunohistochemically stained tissue sections showed a more complex pattern. In all tissues, HP1BP3 is strictly a nuclear protein, although the intranuclear distribution appears to be cell type specific. In some tissues such as the brain, most of the cells had high levels of the protein (Figure 2F). In other tissues, exemplified by the kidney (Figure 2C), the levels of HP1BP3 varied from a strong signal in the distal tubules to much weaker ones in the proximal tubules. Interestingly, HP1BP3 is not expressed in the germ cells as evidenced in the testis (Figure 2E) and the ovaries (Figure 2B). We observed widespread expression of HP1BP3 in embryonic tissue, beginning as early as E6.5 (Figure 2D). This expression was restricted to the embryo itself and HP1BP3 was absent from all trophoblast placental regions. The intra-nuclear distribution of the protein varies with cell type, with some, such as the Purkinje (Figure 2E) presenting a clear enrichment in the dense perinucleolar heterochromatic foci, while in others, such as neurons of the cerebellar granular layer the HP1BP3 protein pattern is mostly diffuse.

In view of the nuclear localization of HP1BP3, we sought to map its nuclear NLS by mutant analysis. To this end we constructed a range of GFP fused fragments of HP1BP3 (Supplementary Figure S2) and expressed them in HeLa cells. The cells were counterstained with DAPI and observed for the localization of the protein. Only fragments containing GD1 or the CTD were restricted to the nucleus. This is particularly interesting, since GD1 does not contain any amino acid motif resembling an NLS. The other globular domains and the NTD were equally distributed in the nucleus and cytoplasm and did not confer nuclear localization.

### HP1BP3 is enriched in heterochromatin in an HP1-dependent manner

As its name implies, HP1BP3 has been shown to interact with heterochromatin protein 1 (HP1), a key mediator of heterochromatin formation and propagation (38,39). Since we observed enrichment of HP1BP3 in heterochromatin *in* *vivo*, we aimed to characterize the nature of this localization also in cultured MEF cells using the heterochromatin markers HP1α or H3K9me3 (Figure 2G). Confocal images show clear co-localization of HP1BP3 with both markers, corroborating enrichment in the dense perinucleolar heterochromatin foci. Consistent with these results, mCherry-HP1BP3 colocalizes in live cells with GFP-HP1α and GFP-HP1β. As expected, a markedly lower colocalization was observed with the non-heterochromatin GFP-HP1γ (Supplementary Figure S2).

HP1 functions by binding to tri-methylated lysine 9 on histone H3 via its chromo-domain and interacting with other proteins via its chromoshadow domain and a cognate PxVxL motif in the binding partners. HP1BP3 contains a canonical HP1 binding motif, PQVKL, located at residues 255–259 between the first two globular domains (Figure 1A) and shown to be essential for the interaction with HP1 (39). Therefore, in order to test the role of HP1 in the localization of HP1BP3, we mutated the central valine (V257) in the sequence to glutamate, a mutation previously shown to abolish the interaction of binding proteins with HP1 (30,40–47). Indeed, this single point mutation led to a dramatic loss of HP1BP3 enrichment in heterochromatin, observed in MEF cells expressing GFP-HP1BP3^V257E^ (Figure 2H-I).

### Chromatin binding dynamics of HP1BP3 are similar to those of H1

**Figure 3.**
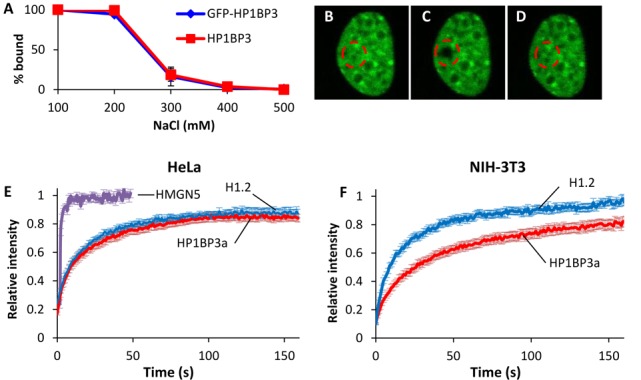
HP1BP3 chromatin binding dynamics are similar to those of histone H1. (**A**) Salt extraction of endogenous and GFP fused HP1BP3. Cells stably expressing GFP-HP1BP3^WT^ were lysed in increasing salt concentration and GFP tagged versus endogenous HP1BP3 levels in soluble/insoluble fractions were assayed by western blot. Data presented as percent bound to chromatin at each concentration, ±SEM. (**B**–**D**) FRAP series of HeLa cells expression GFP-HP1BP3^WT^, with bleached area shown (dashed circle). Panels represent prebleach (B), bleach (C) and plateau level (D). (**E**) FRAP of HeLa cells expressing GFP-HP1BP3, GFP-H1.2 or YFP-HMGN5. Data are presented as mean ± SEM. (**F**) FRAP of NIH-3T3 cells expressing GFP-HP1BP3 or GFP-H1.2. Data are presented as mean ± SEM.

FRAP has been used extensively in recent years to analyze the chromatin binding dynamics of nuclear proteins. In order to apply this method to HP1BP3 we stably transfected HeLa cells with N-terminal GFP fused to HP1BP3. The time frame for recovery in FRAP varies widely among the different chromatin binding proteins, from a few seconds for many transcription factors and members of the HMG family of proteins, to minutes for histone H1 subtypes. To place HP1BP3 within the context of these architectural proteins, we transfected cells with YFP-HMGN5 or GFP-H1.2 as representatives of these two groups. We first compared the association of the endogenous and ectopically expressed HP1BP3 to DNA by extracting them from chromatin under increasing salt concentrations. Figure 3A shows that the extraction profiles of the tagged and endogenous proteins were identical, suggesting that the GFP moiety did not significantly affect the interaction of the protein with chromatin.

FRAP analyses of HeLa cells stably expressing GFP-HP1BP3a, GFP-histone H1.2 or YFP-HMGN5 (Figure 3B–E) showed remarkably similar FRAP curves for HP1BP3 and H1.2, with *τ1/2* values of 14.1 ± 0.9 and 11.3 ± 1.5 s respectively, and immobile fractions of 12.8 ± 2.2 and 11.2 ± 2.2%. HMGN5 behaved as has been previously described (32), with a *τ1/2* of 0.54 ± 0.2 s and no immobile fraction. No difference was observed in the FRAP curves of HP1BP3a and HP1BP3b (Supplementary Figure S1C), and HP1BP3a was used for all subsequent experiments. The relatively slow recovery of HP1BP3 is not restricted to HeLa cells and was observed in NIH-3T3 cells as well (Figure 3F). Interestingly, while the recovery rates of H1.2 were identical in both cell types, HP1BP3 recovery time was doubled in the 3T3 cells, with a τ_1/2_ of 27.8 ± 2.5 s (*P* < 0.001), suggesting cell-specific behavior.

**Figure 4.**
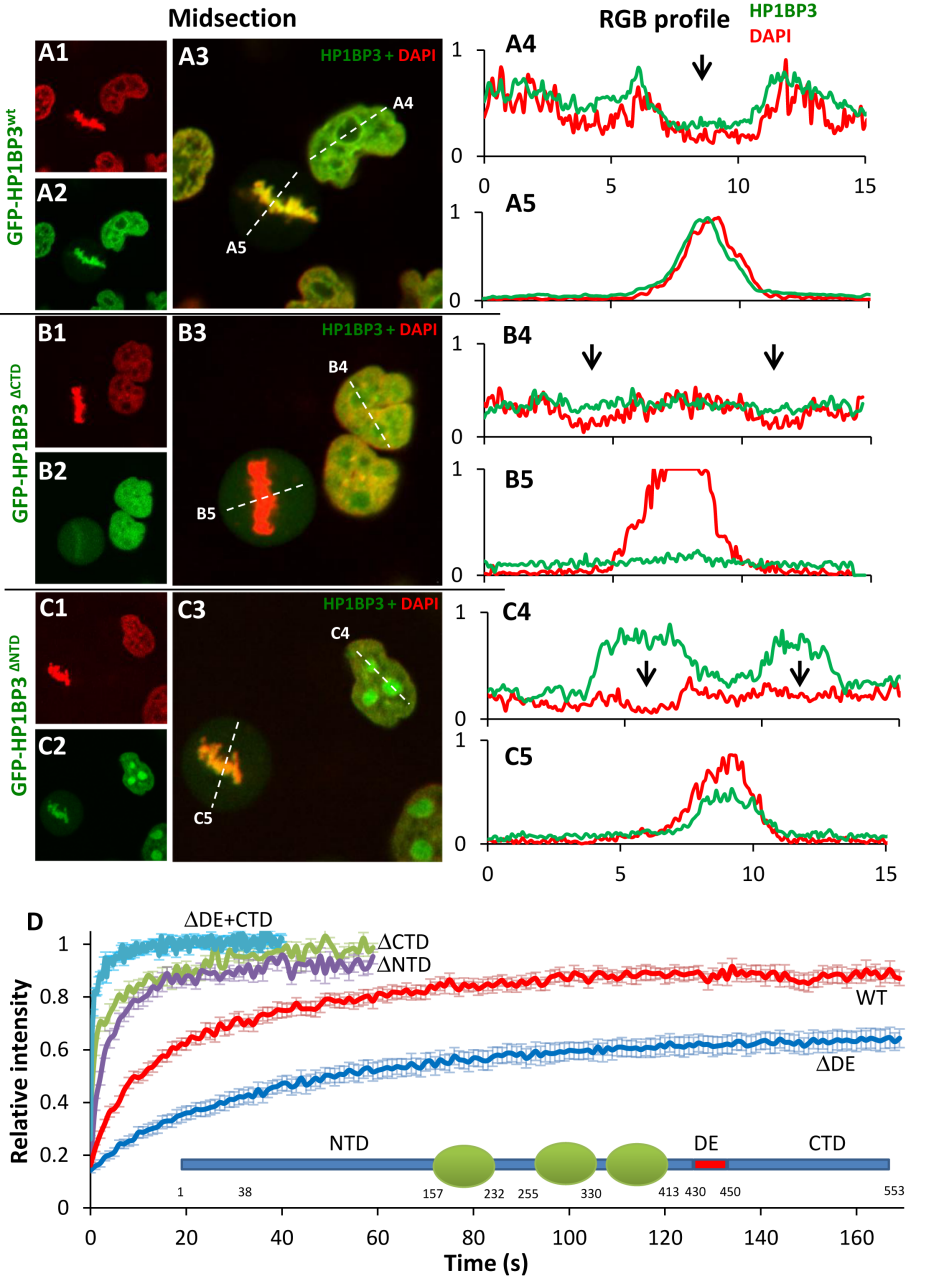
Analysis of binding determinants in HP1BP3. (**A1**–**C5**) HeLa cells stably expressing GFP-HP1BP3^WT^ (A1–A3), GFP-HP1BP3^ΔCTD^ (B1–B3) or GFP-HP1BP3^ΔNTD^ (C1–C3) were stained with Hoechst 33342 (pseudo-red) and observed by live using confocal microscopy. RGB profiles were measured for the green and red channels of each construct in interphase (A4, B4, C4) and metaphase (A5, B5, C5) cells. Profiles of GFP-HP1BP3^WT^ (green line) and Hoechst (red line) in both cell cycle phases (A4 and A5) follow similar patterns, including relative nucleolar exclusion (black arrow in A4), suggesting co-localization. In contrast, GFP-HP1BP3^ΔCTD^ remains diffuse regardless of cell cycle stage and is not excluded from nucleoli (black arrows B4). GFP-HP1BP3^ΔNTD^ shows an inverse distribution in interphase, with high nucleolar enrichment (C4), while retaining normal chromatin binding in metaphase (C5). (**D**) FRAP curves of HeLa cells expressing GFP-HP1BP3^WT^, GFP-HP1BP3^ΔCTD^, GFP-HP1BP3^ΔNTD^, GFP-HP1BP3^ΔDE^ or GFP-HP1BP3^ΔDE + CTD^. Deletion of either the CTD or NTD of HP1BP3 dramatically reduces binding to chromatin (*P* < 0.001 for both). Conversely, deletion of the DE region leads to a large increase in binding (*P* < 0.001). Deletion of both the CTD and the DE region abolishes binding entirely. Data are represented as mean ± SEM.

**Figure 5.**
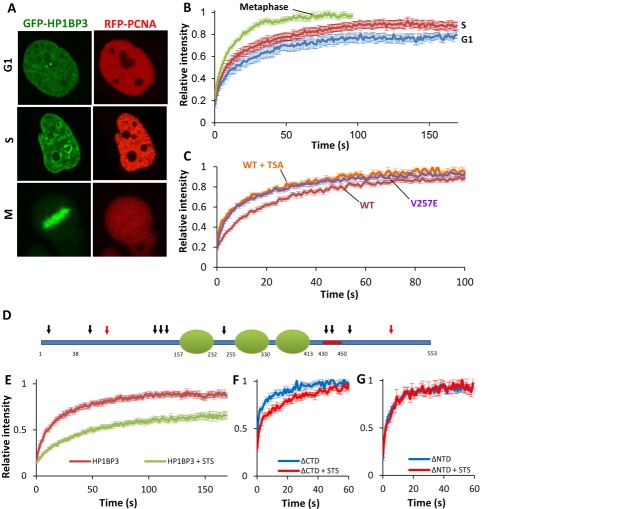
External determinants of HP1BP3 binding to chromatin. (**A** and **B**) Binding of HP1BP3 to chromatin is cell cycle dependent. (A) Cell-cycle stages of HeLa cells expressing GFP-HP1BP3^WT^ (first column) were identified by the subnuclear pattern of co-expressed RFP-PCNA (second column), which is diffuse in G1 and punctate in S phase. Metaphase cells were recognized by the obvious mitotic figures apparent in these cells. (B) Cells were next subjected to FRAP, and results plotted ±SEM. The immobile fraction is significantly reduced in S-phase cells (*P* < 0.05) and τ_1/2_ decreased in M phase (*P* < 0.001). (**C**) FRAP of cells expressing the GFP-HP1BP3^V257E^ mutant or cells expressing the GFP-HP1BP3^WT^ construct treated with TSA. Mutation of the HP1 binding site at V257E in the GFP-HP1BP3^V257E^ led to a two-fold reduction in τ_1/2_ (*P* < 0.001). Treatment of cells expressing GFP-HP1BP3^WT^ with 100 ng/ml TSA for 2 h prior to FRAP led to similar results (*P* < 0.005). (**D** and **E**) The role of phosphorylation in binding of HP1BP3. (D) Schematic representation of phosphorylation sites in HP1BP3. Black arrows represent documented sites, red arrows predicted. (E) Cells expressing GFP-HP1BP3^WT^ were treated with 1 mg/ml Staurosporine or vehicle for 1.5 h. FRAP curves show a dramatic increase in binding following treatment with STS (*P* < 0.001). (**F**) Cells expressing HP1BP3^ΔCTD^ were treated with STS as above and FRAP shows that binding was significantly (*P* < 0.002) increased (**G**) Cells expressing GFP-HP1BP3^ΔNTD^ were treated as above, but this had no effect on binding.

We next explored the roles of the different domains of HP1BP3 in chromatin binding. To this end we created deletion mutants fused to GFP and expressed them in HeLa cells. Live staining with Hoechst showed that, as expected, full length HP1BP3 is restricted to the nucleus; furthermore, HP1BP3 colocalizes with DNA throughout the cell cycle and is relatively excluded from nucleoli (Figure 4, panels A3–5). Visually, the mutant lacking the 20 amino acid long DE region (ΔDE) looked identical to the full length protein (not shown). However, deletion of this region greatly increased chromatin binding (Figure 4D), with the τ_1/2_ doubling to 29.7 ± 2.7 s and the immobile fraction tripling to 34.7 ± 3.9%. (*P* < 0.001 for both). In contrast to the full length and ΔDE mutant, deletion of the CTD abolished the apparent colocalization with DNA and the GFP signal became diffuse throughout the cell cycle (Figure 4B). Furthermore, FRAP analysis revealed that chromatin binding of ΔCTD was dramatically reduced seven-fold with a τ_1/2_ of 2 ± 0.3 s (Figure 4D). Surprisingly, deletion of both the CTD and the DE region had an even stronger effect, essentially abolishing the interaction with chromatin (Figure 4D). Deletion of the NTD led to a redistribution of the protein during interphase and it became highly enriched within nucleoli (Figure 4C3–4); however, unlike the ΔCTD mutant, ΔNTD does colocalize with DNA during metaphase. In terms of chromatin binding parameters, deletion of the NTD had a similar effect to that of the CTD and the τ_1/2_ was reduced to 3.1 ± 0.3 s (Figure 4D). Finally, to assess the potential importance of the PQVKL motif and HP1 binding for the interaction of HP1BP3 with chromatin, we used the GFP-HP1BP3^V257E^ mutant. We found that this single point mutation had a dramatic effect on the binding of HP1BP3 to chromatin and it reduced the τ_1/2_ by almost half, to 8 ± 0.5 s (Figure 5C).

To test the effect of cell cycle on the chromatin binding dynamics of HP1BP3, cells were transfected with RFP-PCNA. The two very distinct nuclear patterns presented by PCNA during G1 and S phases allowed us to discern the current cell cycle phase in live cells (Figure 5A). FRAP analyses suggests that the immobile fraction of HP1BP3 is larger in G1 than in S (22 ± 2.7 and 11.4 ± 3.1%, respectively (*P* < 0.05; Figure 5B). In metaphase cells, easily recognized by the alignment of chromosomes along the mitotic plate, the binding affinity of HP1BP3 was greatly reduced with a τ_1/2_ of 7.8 ± 0.52 s and no immobile fraction.

Phosphorylation of histone H1 has been shown to play a significant role in the modulation of H1 binding to chromatin and recent global phosphoproteomic studies have identified phosphorylation of specific residues in HP1BP3 (48–53) (Figure 5D). To assess the potential effect of HP1BP3 phosphorylation on chromatin binding, cells were treated with 1 μM staurosporine (STS), a known pan-inhibitor of cellular kinases and of H1 phosphorylation specifically (54). Addition of STS 1.5 h prior to FRAP led to a dramatic increase in chromatin binding of HP1BP3, with a τ_1/2_ of 30.3 ± 2.5 and an immobile fraction of 33.7 ± 3% (Figure 5E). This effect could result from inhibition of phosphorylation of HP1BP3 itself or other unknown protein targets involved HP1BP3 binding. Following this observation we attempted to determine which region of HP1BP3 is involved in the STS mediated effect by treating cells expressing the ΔNTD and ΔCTD mutants with STS. Consequently, the τ_1/2_ of ΔCTD tripled from 2 ± 0.3 to 5.9 ± 0.9 (Figure 5F), while the ΔNTD was not affected (Figure 5G), suggesting that relevant phosphorylation sites are located in the NTD. Finally, the effect of core histone acetylation was measured by adding TSA to cells 2 h before FRAP (Figure 5C). Treatment with TSA led to a reduction in the τ_1/2_ (10 ± 0.8 s, *P* < 0.003) suggesting that histone acetylation reduces the chromatin residence time of HP1BP3.

### Depletion of HP1BP3 affects gene expression but does not alter global chromatin organization

**Figure 6.**
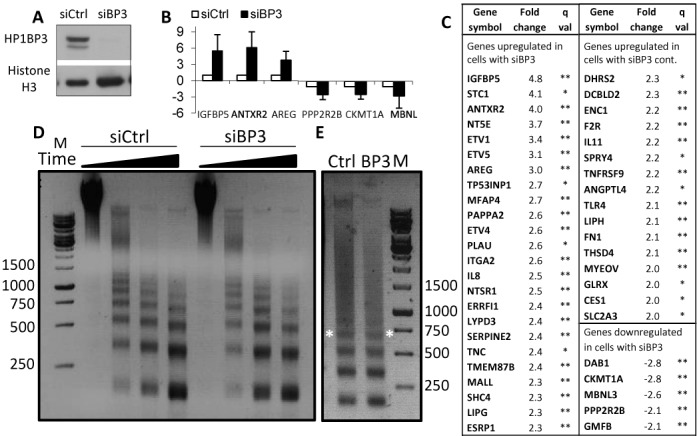
Transcriptional regulation by HP1BP3. HeLa cells were transfected in triplicate with siRNA using either negative control or HP1BP3 specific oligos. Protein and RNA were isolated from cells 72 h post transfection. (**A**) Protein samples were analyzed by western blot using anti HP1BP3 and anti-histone H3 for reference. Note that there was complete knockdown of HP1BP3 at this time. (**B**) The RNA was treated with DNase1, reverse transcribed and hybridized to Affymetrix human gene ST1.0 microarrays. Analysis was performed using Partek 6.0 software. The expression of 230 genes was changed >1.5-fold, with the majority being upregulated in the siHP1PB3 samples. Semi-quantitative Real-time PCR was used to validate upregulated genes and downregulated genes. (**C**) The 40 genes with greater than two-fold change in expression are shown. (**D**) Following the same siRNA treatment, cells were were permeabilized and treated with MNase for increasing amounts of time (2, 5, 10, 15 min). After purification, DNA was run on 1.5% agarose gel and observed for nuclease sensitivity. White shadow at 1500 bp is due to the loading dye. (D) For nucleosome repeat length, cells were treated as in D, but incubated for 10 min only. Tetra-nucleosomes are marked with white asterisks.

To explore the potential role of HP1BP3 in transcriptional regulation we performed microarray analysis of gene expression in HeLa cells following HP1BP3 ablation using siRNA (Figure 6A). As was observed for members of the histone H1 gene family (55,56), a very limited subset of genes was affected by knockdown of HP1BP3. Real-time PCR analyses confirmed the results for six gene products with relatively high fold changes (Figure 6B). Only 45 genes have expression differences (*q* ≤ 0.1) of two-fold or more in si*HP1BP3* cells compared with cells treated with siControl (Figure 6C). Among these 45 genes, there were 40 with increased expression and only five with decreased expression, indicating that ablation of HP1BP3 had a mostly positive effect on gene expression. Reducing the cutoff fold-change to 1.4 increased the number of affected genes to 383, with 271 upregulated and only 112 downregulated (Supplementary Table S3). Analysis of the 383 affected genes using The Database for Annotation, Visualization and Integrated Discovery (DAVID) (57) showed no significantly enriched biological processes or pathways.

Since HP1BP3 is enriched in heterochromatin, we explored the possibility that the effect on transcription could result from a global reduction in chromatin compaction. To address this question, HeLa cells treated with control or HP1BP3-specific siRNA were permeabilized using lysolecithin and treated with MNase for increasing amounts of time. Figure 6D shows that upon depletion of HP1BP3, no difference in the sensitivity of chromatin to MNase digestion was observed. Furthermore, HP1BP3 depletion had no effect on the nucleosome repeat length (Figure 6E). Taken together, these results suggest that the global compaction of chromatin was not altered under these circumstances.

### HP1BP3 is essential for postnatal viability and growth in mice

**Figure 7.**
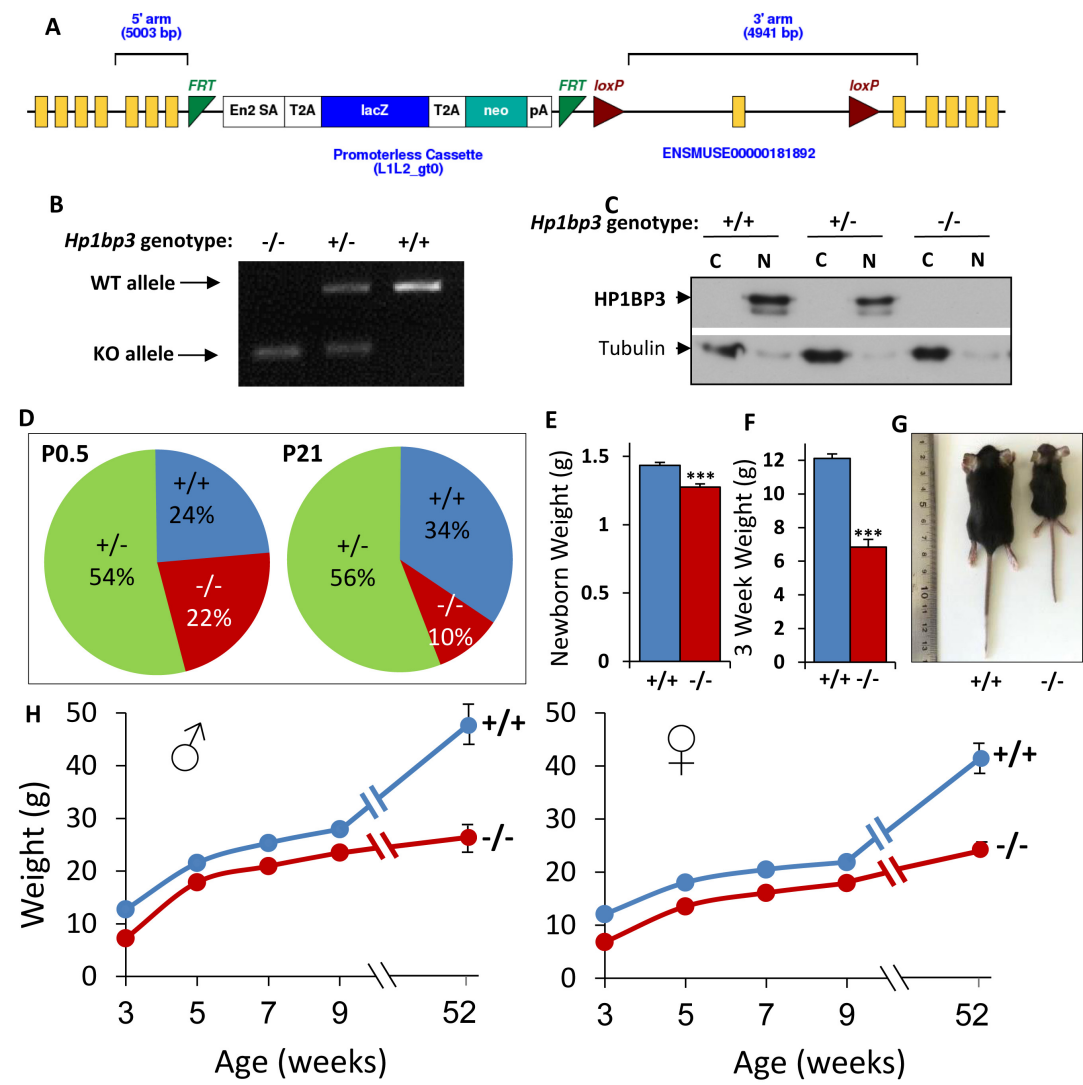
HP1BP3 is essential for mouse viability and growth. (**A**) Design of the gene trap used by the EUCOMM consortium for the creation of the *Hp1bp3*^−/−^ mice, adapted from the EUCOMM website. (**B**) Genotype determination using PCR. All mice are genotyped using genotype-specific primers, allowing detection of the WT and KO alleles. (**C**) HP1BP3 protein is absent from KO mouse cells. MEF cells were isolated from e14.5 embryos, proteins were separated into nuclear (N) and cytoplasmic (C) fractions and samples were analyzed by western blot for HP1BP3 and tubulin as a loading control. Both splice variants are apparent in the *Hp1bp3*^+/+^ mice. A 50% reduction in the intensity of these bands is apparent in the *Hp1bp3*^+/−^ mice, and they are completely absent from the *Hp1bp3*^−/−^ samples. (**D**) Postnatal survival of *Hp1bp3*^−/−^ mice. Whole litters from heterozygous crosses were genotyped either at P0.5 (A1, *n* = 130) or at P21 (A2, *n* = 780). (**E**) Pups from heterozygous litters were weighed at P0.5 and a 10% difference was observed between *Hp1bp3^−/-^* and *Hp1bp3^+/+^* mice. *n* = 30 for each group, *P* < 0.001, data are presented as mean ± SEM. (**F**–**H**) *Hp1bp3^−/−^* mice are dwarfs throughout their life. (F) Weanlings from heterozygous litters were weighed at P21, and a 45% difference was observed between *Hp1bp3^−/^*^−^ and *Hp1bp3^+/+^* mice. *n* > 25 for each group, *P* < 0.001, data are presented as mean ± SEM. (G) Three*-*week old mice were photographed at weaning. (H) Male and female mice from heterozygous litters were weighed bi-weekly from weaning (3 weeks) to 9 weeks, then again at 1 year. *n* > 6 for each data point; *P* < 0.001 for all ages for both sexes; data are presented as mean ± SEM.

We next explored the potential *in* *vivo* role for HP1BP3. To this end, we obtained a strain of *Hp1bp3* knockout mice from the EUCOMM. In this strain, the *Hp1bp3* locus is modified by the insertion of a cassette, containing a splice acceptor, the β-galactosidase gene (LacZ) followed by a polyadenylation signal and the gene for neomycin phosphotransferase (Neo), all separated by T2A cleavage-sequences between exons 7 and 8 of *Hp1bp3* (Figure 7A). Transcription of the disrupted allele is expected to produce a transcript containing exons 1–7, which, if translated, would correspond to the first 150 amino acids of HP1BP3. The disrupted *Hp1bp3* allele will herein be designated as *Hp1bp3^−/−^*. The genotype was confirmed by PCR (Figure 7B) and full-length HP1BP3 protein is undetectable in *Hp1bp3^−/−^* MEF cells and mouse tissues (Figure 7C). We also found no evidence of a truncated protein (not shown) suggesting that if translated, this product undergoes rapid degradation and, therefore, this can be considered a null allele. To determine the viability of homozygous null (*Hp1bp3^−/−^*) mice, *Hp1bp3^+/−^* mice were intercrossed and the genotype distribution of 784 weanlings was determined (Figure 7D). At weaning, the *Hp1bp3* genotypes were not distributed 1:2:1, as expected for Mendelian inheritance of the nonfunctional *Hp1bp3^−/−^* allele. Instead, the ratio of *Hp1bp3^+/^*^+^ to *Hp1bp3^+/−^* to *Hp1bp3^−/−^* in 784 weanlings was 3.5:5.8:1, indicating that the *Hp1bp3^−/−^*genotype causes embryonic or early post-natal lethality (*P* < 0.0001). Furthermore, the ratio of males to females among the *Hp1bp3^−/−^* weanlings was 1:1.6 respectively, suggesting that the lethality is sexually dimorphic (*P* < 0.02). Interestingly, the *Hp1bp3^+/−^* genotype was also slightly underrepresented (*P* < 0.01), suggesting dosage dependence of the phenotype.

To find out when the lethality occurs, the genotypes of 130 newborn pups from *Hp1bp3^+/−^* crosses were determined (Figure 7D). The ratio of *Hp1bp3^+/^*^+^ to *Hp1bp3^+/−^* to *Hp1bp3^−/−^* was 1.1:2.4:1, indicating that the observed lethality is post-natal. In an attempt to understand the cause of this lethality we analyzed the blood of newborn pups, but found no differences in any of the measured parameters (Supplementary Table S2), so that the cause of death is currently unknown.

Both male and female *Hp1bp3^−/−^* mice that survive to weaning are fertile and have a normal life span (not shown). However, already at birth they are smaller than their littermates and weigh 10% less (*P* < 0.0001)(Figure 7E). This growth retardation is further exacerbated in those *Hp1bp3^−/−^* mice that survive into adulthood (Figure 7F–H). By the age of 3 weeks, both male and female *Hp1bp3^−/−^* mice weigh almost 50% less than their littermates (Figure 7F). This gap decreases to 20% by 5 weeks of age and remains stable throughout their young adult life. Interestingly, at the age of one year, the *Hp1bp3^−/−^* mice again weigh 50% less than their normal littermates due to the age-related increase of the latter (Figure 7H).

Since HP1BP3 is a structural chromatin protein, we considered that impaired chromatin compaction or heterochromatin organization could account for the observed lethality and growth defects of *Hp1bp3^−/−^* mice. To test this hyposthesis, we cultured MEF cells from *Hp1bp3^+/^*^+^ and *Hp1bp3^−/−^* embryos, and assessed a number of parameters. First, staining of fixed cells with anti-HP1α or anti-H3K9me3 showed apparently unperturbed organization of heterochromatin (Supplementary Figure S4A and B). Furthermore, consistent with the results in HeLa cells (Figure 6D and E), we observed no changes in the sensitivity of chromatin from *Hp1bp3^−/−^* MEFs to MNase digestion (Supplementary Figure S4C) and no change in the nucleosome repeat length (Supplementary Figure S4D). Finally, we found no difference in the size of nuclei from *Hp1bp3^−/−^* MEF cells. Taken together, these results suggest that the lethality and growth retardation of *Hp1bp3^−/−^* mice are not due to global defects in chromatin organization.

## DISCUSSION

In this study we show that HP1BP3 evolved from the linker histone H1 gene family and that it retains many of the family's structural and functional characteristics. The linker histones of higher organisms have a tripartite structure consisting of a central globular core flanked by a long unstructured basic C-terminal tail and a shorter N-terminal extension. FRAP studies revealed that in live cells, the binding of H1 to nucleosomal DNA is a multistep process that involves cooperation between the globular and CTDs (58–62). Thus, deletion of either the CTD (63) or the GD (59) dramatically reduce the affinity of H1 to chromatin in live cells. Similar to all H1 variants, HP1BP3 also has a tri-partite structure, with an extended triple globular region flanked by a long unstructured basic C-terminal tail and a long N-terminal extension. Our analysis of HP1BP3 deletion mutants using FRAP shows that the different regions share functional roles with those of H1 subtypes. We find that deletion of the positively charged CTD greatly reduces the affinity of HP1BP3 for chromatin, as is evident from the seven-fold decrease in the FRAP recovery time. Conversely, when the CTD alone was fused to GFP, FRAP recovery was even faster (τ_1/2_ of ∼0.25 s, not shown). These results suggest that both the GD and the CTD are critical for avid binding to nucleosomal DNA, but only through their mutual cooperation. In this respect we note that proper cooperative binding of H1 to nucleosomes has been shown to induce chromatin compaction and a recent study found that HP1BP3 can compact chromatin *in vivo* (64).

Recent work has suggested that the N-terminal region of H1 plays a part in the chromatin binding process, probably by mediating the proper positioning of the GD relative to the nucleosome. Swapping of the NTDs from H1.0 and H1.2 led to swapping of their chromatin binding characteristics (65) and deletion of the H1.4 NTD led to a decrease in affinity and loss of specificity for nucleosomal DNA (66). Deletion of the NTD of HP1BP3 also decreased the affinity of the protein for chromatin. Interestingly, this deletion had the added effect of redistributing the protein within the nucleus, with very high enrichment in the nucleoli.

FRAP has been used extensively to explore the chromatin binding dynamics of nuclear proteins in live cells. These experiments have shown that the chromatin binding proteome is far more dynamic than was originally anticipated. Particularly interesting was the demonstration that linker histones are not stably bound to nucleosomes, but rather continuously exchange between chromatin sites (31,67). Even so, FRAP of H1 showed it to move much more slowly in nuclei than most other proteins, including transcription factors (4,68) and other architectural proteins such as members of the HMG family (2,32). Our results indicate that HP1BP3 traverses the nucleus at similar rates to those of H1, far more slowly than HMGN5. The mobility of nuclear proteins observed by FRAP was recently found to be determined primarily by their affinity for nucleosomal DNA (68,69), suggesting that like H1, HP1BP3 binds to nucleosomes with high affinity. Further supporting this conclusion is the observation by a previous study that GD1 of HP1BP3 can bind to nucleosomes at their entry and exit sites *in* *vitro* (39). Interestingly, we find that GD1 also shares NLS functionality with the GD of H1 (70).

Further similarities to histone H1 subtypes were found in the effects of the cell cycle and post-translational modifications on HP1BP3 binding to chromatin. Cell cycle–related chromatin decondensation and phosphorylation of H1 have been shown to be associated with changes in the intranuclear mobility of the protein, and inhibition of H1 kinases decreases H1 mobility in living cells (71–74). FRAP experiments have also demonstrated that exposure of cells to histone deacetylase inhibitors reduces the binding of H1 to chromatin (31). We find that HP1BP3 follows these same patterns, showing increased mobility with progression of the cell cycle and following treatment with TSA and decreased mobility in response to kinase inhibition.

In addition to the common binding determinants shared with other H1 family members, HP1BP3 also contains an acidic stretch separating the third GD from the CTD. This region, which we called the DE region, is unique to HP1BP3 within the H1 gene family, but is typical of many nuclear chaperones. In these, the DE sequence plays an interesting dual role, interacting with core histones on one hand and modulating DNA binding on the other (75–78). Our results suggest that this region may play a similar role in HP1BP3, since deletion of the DE domain dramatically increased the residence time of HP1BP3 on chromatin, probably due to the removal of electrostatic interference. On the other hand, whereas the CTD was necessary for HP1BP3 binding to chromatin, simultaneous deletion of both the CTD and DE regions decreased binding below that observed under the CTD deletion alone, perhaps due to the loss of interaction with the core histones.

Of the 11 mammalian H1 subtypes described to date, one is expressed in oocytes only, three are sperm specific and the rest are expressed in somatic cells. Our analysis of HP1BP3 expression patterns in murine tissues showed that the protein is ubiquitously present in somatic cells and clearly absent from gonadal cells, suggesting a somatic classification. In all observed tissues and cells, HP1BP3 was restricted to the nucleus. Previous studies of H1 proteins using specific antibodies in fixed cells (79), live imaging of GFP fused subtypes (80) and more recently DamID sequencing (81), all showed that the different H1 subtypes have non-random intra-nuclear distributions and differ in their preferences for nuclear regions. Our immunohistochemical results indicate that the intra-nuclear distribution of HP1BP3 *in vivo* is also non-random and we observed strong enrichment in dense heterochromatin foci in several cell types. The higher concentration in heterochromatic regions is in agreement with recently published data showing high enrichment of HP1BP3 in the heterochromatin of 293T cells (64). In cultured MEF cells, the enrichment of HP1BP3 in heterochromatin was found to be HP1 dependent. Interestingly, the heterochromatin enrichment of HP1BP3 was not abolished entirely by mutating the PQVKL motif. A similar observation was also noted for another HP1 binding protein, ATRX (41), suggesting that targeting to heterochromatin may involve additional interactions other than HP1. In addition to affecting the localization of the protein, mutating the PQVKL motif also led to a significant reduction in the residence time of HP1BP3 on chromatin, suggesting that binding of HP1 stabilizes the interaction with chromatin. This is surprising considering the rather dynamic nature of HP1 itself (30). It is interesting to note that HP1 has been shown to interact with histone H1.4 (82) and H1b (83), but the role of this interaction in the binding dynamics of H1 have not been studied. Furthermore, since these H1 subtypes lack the PxVxL motif, these interactions are not mediated by the chromoshadow domain of HP1.

The potential involvement of histone H1 in transcriptional regulation has always been of interest due to the key role of H1 in chromatin organization. A number of studies have shown that deletion of a single H1 subtypes can lead to changes in the expression levels of surprisingly small numbers of genes (21,55–56). Our results suggest that, when knocked down in HeLa cells, HP1BP3 follows a similar pattern, with 383 genes showing altered expression. All the changes in transcription levels were highly significant; however, the expression levels of most of the affected genes changed only moderately (between 1.4- and 2-fold); <15% of the genes were changed by more than two-fold. It is interesting to note that only 12 of these genes appear in a proteomic analysis of HEK-293T cells with reduced HP1BP3 (64). This apparent cell-type specificity, together with the observed lack of enrichment in specific biological processes, suggests a role for HP1BP3 as a chromatin architectural protein capable of modulating gene transcription rather than a specific *trans*-activating factor (5). It should be noted that due to the use of a gene oriented microarray assay, changes in the expression of non-coding regions under HP1BP3 knockdown conditions may have been overlooked and future work should shed light on the role of HP1BP3 in regulating transcription of these novel targets.

A key shared characteristic of all H1 subtypes is their ability to bind nucleosomes, leading to compaction of DNA at the entrance and exit sites, essentially adding ∼20 bp of DNA to each nucleosome (7). Hayashihara *et al*. (39) previously found that GD1 of HP1BP3 can indeed bind to nucleosomes *in vitro* leading to an increase in the length of DNA protected by the nucleosome. Therefore, to complement our FRAP experiments we tested the effect of ablation of HP1BP3 on nucleosome spacing *in vivo* using MNase digestion of chromatin from permeabilized HeLa or MEF cells and found no change in the nucleosome repeat length (NRL) in either case. In this regard, it is important to note that a quantitative analysis of the metaphase chromosomal proteome in HeLa cells found no more than one HP1BP3 molecule for every 100 molecules of total H1 (84). Thus, if HP1BP3 indeed binds to nucleosomes as part of the total H1 complement, ablation of the protein should reduce the overall H1 level by <1% and is unlikely to affect the NRL due to compensation by other H1 subtypes. Similar compensation underlying a lack of NRL change was also observed even upon depletion of highly abundant H1 subtypes (65).

The severe phenotype of the HP1BP3 knockout mouse model was not expected when viewed in the framework of other H1 knockout results. Targeted mutation of single somatic subtypes of H1 failed to cause any observable phenotype (23,24), most likely due to compensatory upregulation of other subtypes, thereby minimizing changes in the total H1 levels. Consequently, it was necessary to simultaneously ablate the expression of no less than three somatic subtypes in order to effectively reduce the global levels of H1 in the mouse cells, thereby causing embryonic lethality during mid-gestation (22). These results suggest that linker histones are indispensable for murine development, but individual subtypes do not have any critical non-redundant functions. Apparent exceptions to this rule are the gonadal H1 subtypes H1.7 and H1.8, the lack of which appear to cause male and female sterility respectively (15,85). However, these phenotypes are probably due to the inability of somatic subtypes to compensate due to their absence in from the relevant cells. In striking contrast, the partial postnatal lethality and adult dwarfism of mice lacking HP1BP3 indicate that in spite of HP1BP3 being a somatic protein, other H1 subtypes cannot compensate for its loss.

The evolutionary history of histone H1 presents an intriguing example of progressive complexity. The earliest members of this family were simple, basic proteins that bound to linker DNA (29). Fusion of this early sequence to that of a winged helix globular domain added a first layer of complexity to the new tri-partite H1, allowing greater specificity for nucleosomal DNA. As metazoan organisms evolved, another level of complexity was added through gene duplication and the creation of sub-types, culminating in the 11 member mammalian histone H1 gene family (7,8). While the number of subtypes has increased dramatically since the appearance of the tri-partite H1, the structure itself has remained surprisingly unchanged. We now find that HP1BP3 represents a striking exception to this rule, adding yet another level of complexity to the vertebrate H1 gene family by tripling the number of globular domains. Internal duplication of domains within genes is a common pattern of adding complexity over evolution and the proportion of genes with internal duplication grows with evolutionary complexity (86). Collectively, our results suggest that HP1BP3 is a unique and essential chromatin binding protein closely related to histone H1, with novel and non-redundant physiological roles. Further studies will attempt to unravel the molecular basis for the impact of loss of HP1PB3 on gene expression in mice lacking this architectural nuclear protein.

## SUPPLEMENTARY DATA

Supplementary Data are available at NAR Online.

SUPPLEMENTARY DATA
