# Supplementary material for: HP1BP3 is a novel histone H1 related protein with essential roles in viability and growth
Source: Nucleic Acids Res. 2015 Feb 8;43(4):2074–90. doi: 10.1093/nar/gkv089 (PMC4344522; doi:10.1093/nar/gkv089)
Supplement: SUPPLEMENTARY DATA [file supp_43_4_2074__index.html]

HP1BP3 is a novel histone H1 related protein with essential roles in viability and growth — HP1BP3 is a novel histone H1 related protein with essential roles in viability and growth — SUPPLEMENTARY DATA 

# HP1BP3 is a novel histone H1 related protein with essential roles in viability and growth

## SUPPLEMENTARY DATA

**Files in this Data Supplement:**

- SUPPLEMENTARY DATA
- SUPPLEMENTARY DATA
