# Supplementary material for: HP1BP3 is a novel histone H1 related protein with essential roles in viability and growth
Source: Nucleic Acids Res. 2015 Feb 8;43(4):2074–90. doi: 10.1093/nar/gkv089 (PMC4344522; doi:10.1093/nar/gkv089)
Supplement: SUPPLEMENTARY DATA [file supp_gkv089_nar-02307-m-2014-File003.pdf]

A

```
GD1  160 RPKMDAILTEAIKACFQKSGASVVAIRKYIIHKYPSLELERRGYLLKQALKRELNRGVIKQ---VKGKGASGSFVVV 233
H1.8  52 HPPVLRMVLEALQAGEQRRGTSVAAIKLYILHKYPTVDVLRFKYLLKQALATGMRRGLLARPLNSKARGATGSFKLV 128
         :* :  :: **::*  *: *:**.**: **:****:::: *  *******    :.**:: :     *.:**:*** :*

GD2  218 QVKLEDVLPLAFTRLCEPKEASYSLIRKYVSQYYPKLRVDIRPQLLKNALQRAVERGQLEQ-ITG—KGASGTFQLK  291
H1.8  52 HPPVLRMVLEALQAGEQRRGTSVAAIKLYILHKYPTVDVLRFKYLLKQALATGMRRGLLARPLNSKARGATGSFKL  127
         :  :  ::  *:     : : :* : *: *: : **.: *     ***:**  .:.** * : :..  :**:*:*:*

GD3  300 GGSLMEYAILSAIAAMNEPKTCSTTALKKYVLENHPGTNSNYQMHLLKKTLQKCEKNGWMEQ-IS—GKGFSGTFQLC 374
H1.8  52 HPPVLR-MVLEALQAGEQRRGTSVAAIKLYILHKYPTVDVLRFKYLLKQALATGMRRGLLARPLNSKARGATGSFKL 127
           .::.  :*.*: * :: :  *.:*:* *:*.::* .:     :***::* .  :.* : : :.  .:* :*:*:*
```

B

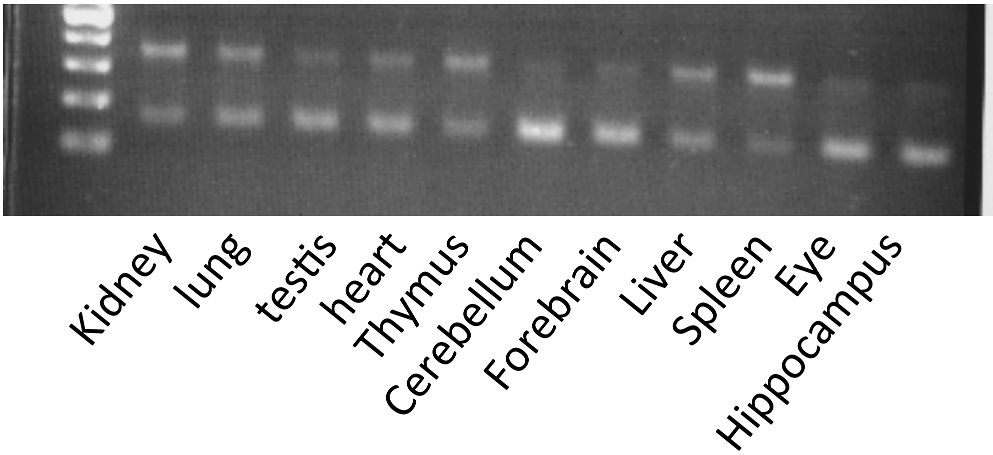

C

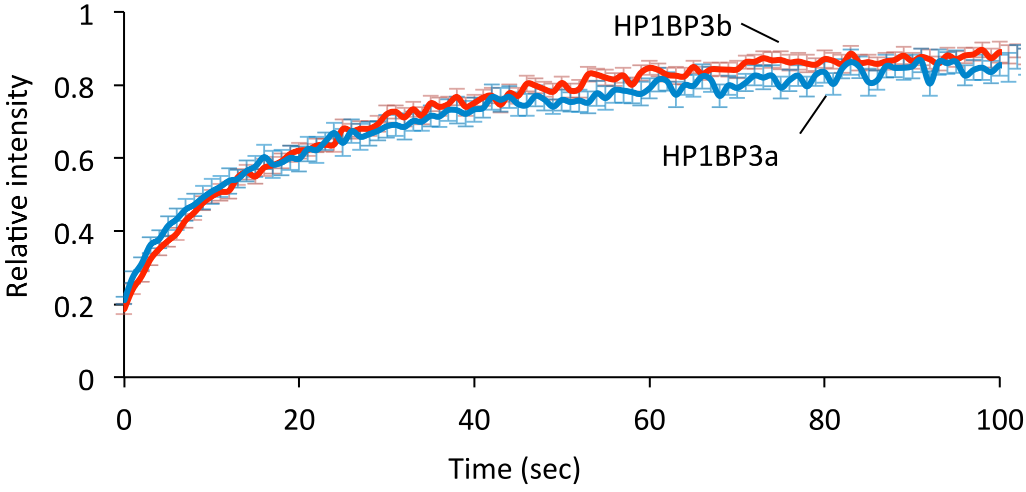

Figure S1: Sequence alignment and alternative splicing of HP1BP3. (A) Alignment of the GDs of HP1BP3 with the histone H1.8 globular domain using ClustalW2 (B) RT-PCR analysis of RNA from various mouse tissues using primers that span exons I to III shows tissue specific splicing patterns for HP1BP3. (C) FRAP of HeLa cells expressing GFP-HP1BP3a or GFP-HP1BP3b. Data are presented as mean ± SEM.

Figure S1. Garfinkel *et al*

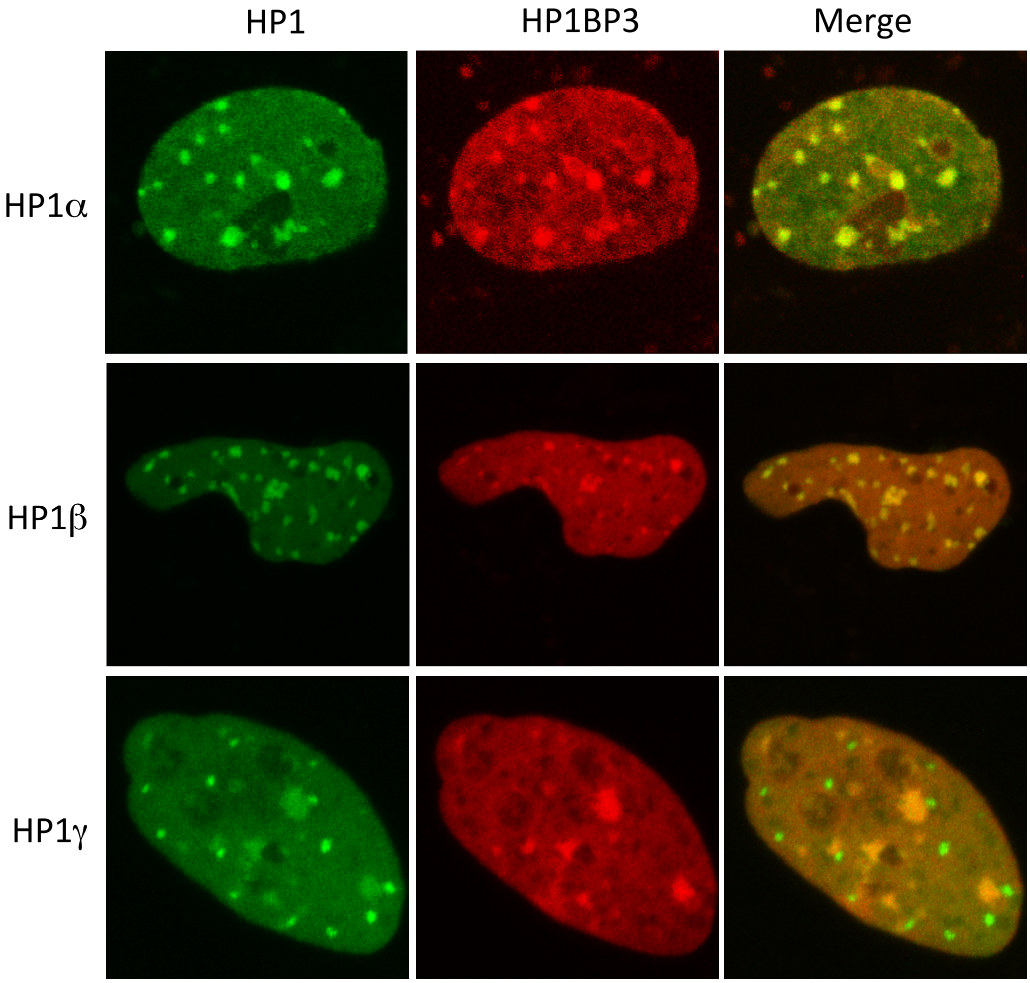

Figure S2: Colocalization with HP1 subtypes. mCherry-HP1BP3 was expressed together with GFP fused HP1α, HP1β or HP1γ in MEF cells. Confocal imaging shows enrichment of HP1BP3 in pericentric heterochromatin together with HP1α and HP1β, and to a lesser extent with HP1γ.

Figure S2. Garfinkel *et al*

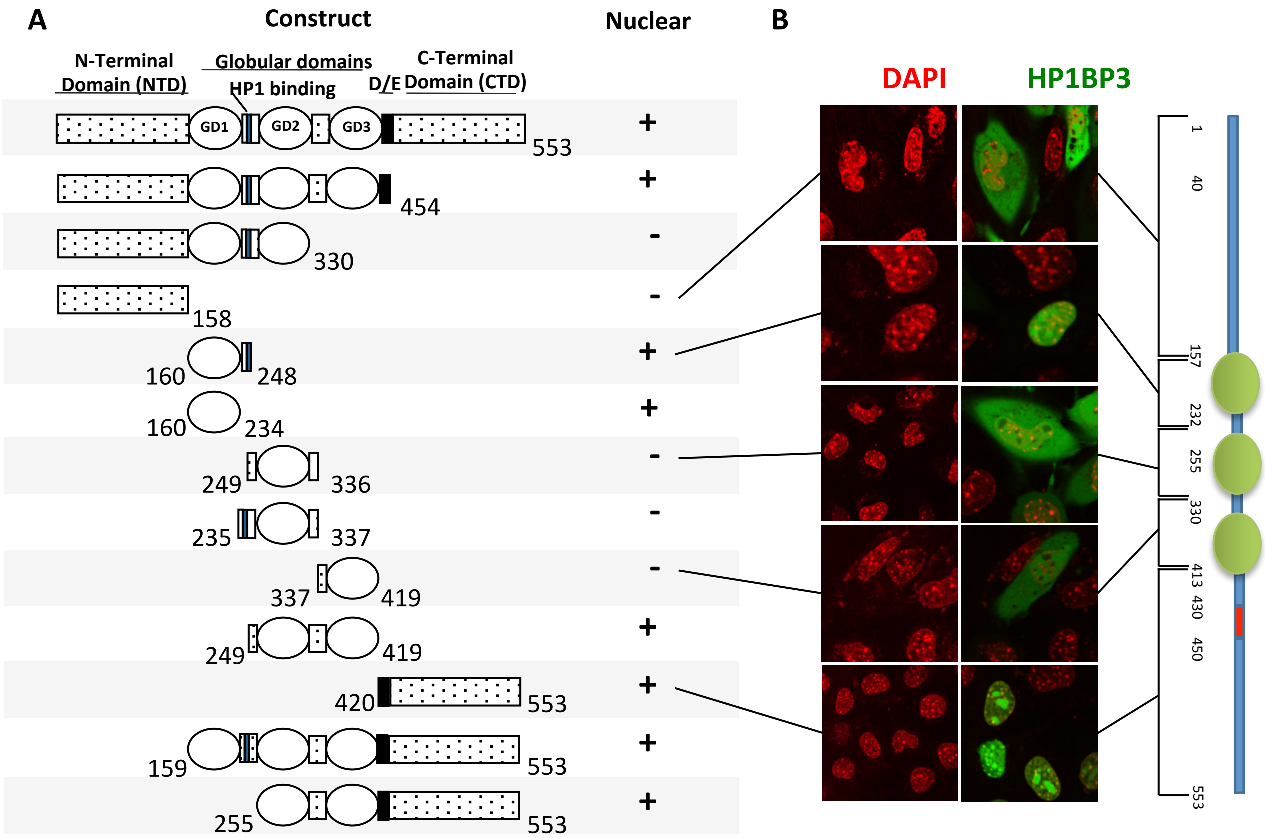

Figure S3: Nuclear Localization. (A) The sequence of HP1BP3a was dissected into fragments, and these were fused to N-terminal GFP and transfected into HeLa cells. Schematic representations with amino acid start and stop numbers are shown. Cells were then analyzed for nuclear or cytoplasmic localization of the GFP signal. (B) Representative images showing localization of GFP-HP1BP3 fragments (green) relative to DAPI (red).

Figure S3. Garfinkel *et al*

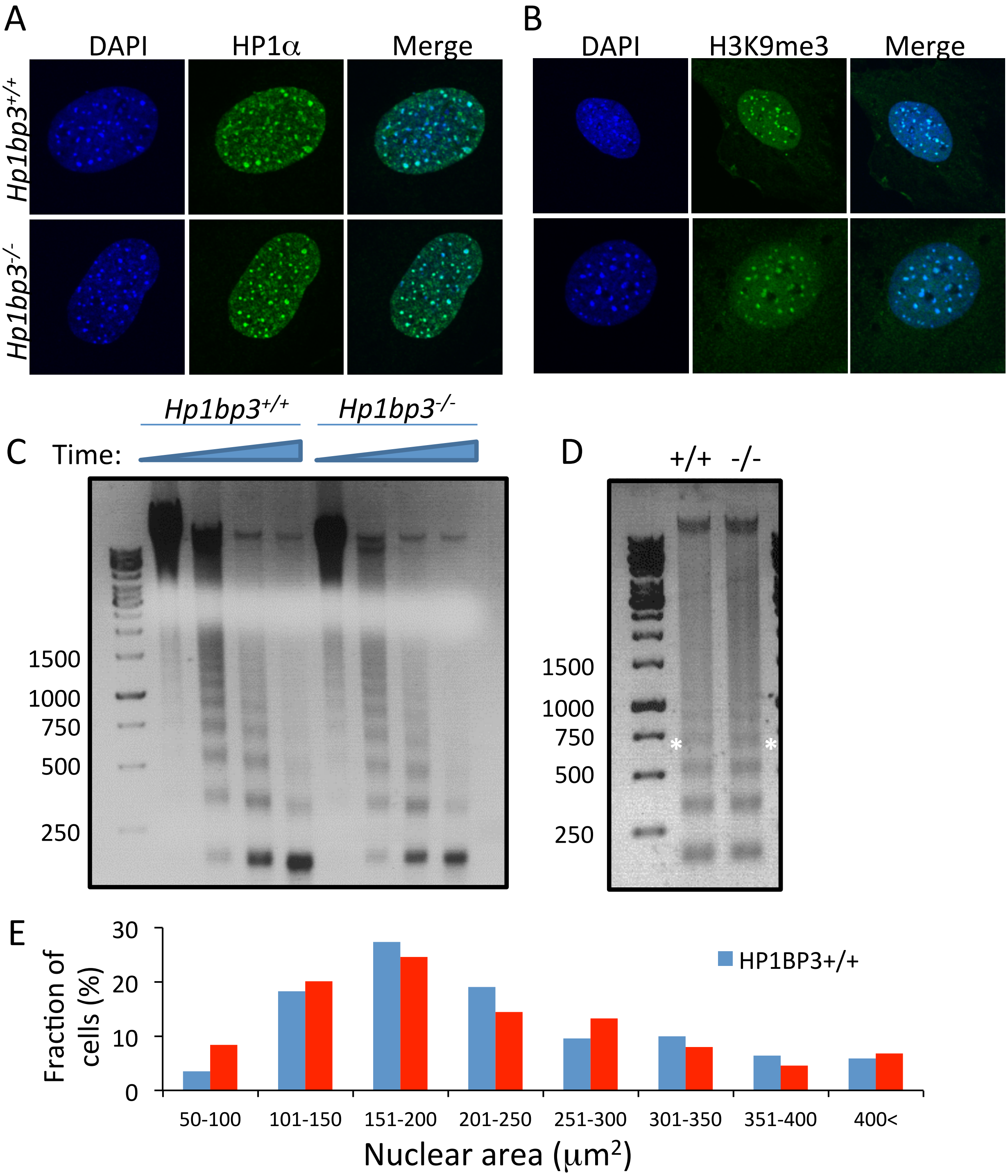

Figure S4: Effect of Hp1bp3 targetted mutation on global chromatin organization. MEF cells from *Hp1bp3* WT or null mice were fixed and stained with DAPI and anti-HP1α (A) or anti-H3K9me3 (B) antibodies to observe pericentric heterochromatin. (C) MEF cells from *Hp1bp3* WT or null mice were permeabilized and treated with MNase for increasing amounts of time (2, 5, 10, 15 min). After purification, DNA was run on 1.5% agarose gel and observed for nuclease sensitivity. (D) MEF cells were treated as in C, but incubated for 10 minutes only and observed for changes in nucleosome repeat length. (E) **.** MEF cells from *Hp1bp3* WT or null mice were fixed and stained with DAPI. The size of nuclei of 300 cells from each genotype were measured.

Figure S4. Garfinkel *et al*

Application	Gene/ region	Primer sequences (5’-3’)
RT-qPCR	IGFBP5	TGTGTACCTGCCCAATTGTG<br>AAGGTGTGGCACTGAAAGTC
	ANTXR2	TTTGTTGAGGCGGCAGTATG<br>ACCTTCTTGGTCTTCCTGCTTC
	AREG	AGGAGAAGCTGAGGAACGAAAG<br>TTGGCAGTGACTCCAATGTG
	CKMT1A	TAACCTGGGCACTGGACTTC<br>GATTTGCCTAGTCGGTCCAA
	MBNL3	CAGCAACAGCACCTGCTAAA<br>ATGCTTTCCCACCCTCTTCT
	PPP2R2B	GTGTGTGTGGAATGGGTCAG<br>ACTTTTCGGGGTTTGAGGAT
RT-PCR	HP1BP3	GCTGGGACTGGAGGACAG<br>TTCCCGGGTAGAATTCACAG
Cloning	pcDNA HP1BP3	GGCCGGTACCAAAATGGCAACTGACATGTCTC<br>GGCCGATATCTTACTTTTTTGTCTTGAAAGACTTCT
	GFP-HP1BP3	GGCCTCCGGACCCAAGATGGACGCAATCTT<br>GGCCGTCGACTTACTTTTTTGTCTTGAAAGACTTC
	ΔNTD	GGCCTCCGGACCCAAGATGGACGCAATCTT<br>GGCCGTCGACTTACTTTTTTGTCTTGAAAGACTTC
	ΔCTD	GGCCTCCGGAATGGCAACTGACATGTCTCAAG<br>GGCCGTCGACTCATAAGCTCCTCTTGGGTGGTGG
	ΔCTD+DE	GGCCTCCGGAATGGCAACTGACATGTCTCAAG<br>GGCCGTCGACTTATGGGCTAGGGTAGTAGGGGAAG

Table S1: List of all primers used in this study.

Table S1. Garfinkel *et al*

Application	Gene/ region	Primer sequences (5’-3’)
	GFP-NTD	GGCCTCCGGAATGGCAACTGACATGTCTCAAG<br>GGCCGTCGACTTACCGTGGGGAGGAAGCCATGG
	GFP-GD1	GGCCTCCGGACCCAAGATGGACGCAATCTT<br>GGCCGTCGACTTAGCCCTTCTTTCTGTTTTTGG
	GFP-GD2	GGCCTCCGGATCGGCTCTGGATCCAGAACC<br>GGCCGTCGACTTACAAGGGCTTCTCCCCTGATT
	GFP-GD3	GGCCTCCGGACTGGGTGGAAGCCTGATGG<br>GGCCGTCGACTTATGGGCTAGGGTAGTAGGGGAAG
	GFP-CTD	GGCCTCCGGAGGAGTTCTATTTCCGAAGAAAG<br>GGCCGTCGACTTACTTTTTTGTCTTGAAAGACTTC
Quickchange	V257E	CTGGATCCAGAACCACAAGAAAAACTGGAAGATGTTCTC<br>GAGAACATCTTCCAGTTTTTCTTGTGGTTCTGGATCCAG
Phusion SDM (5'P)	∆DE	CCACCACCCAAGAGGAGCTTAC<br>AGAGCCACCGGATTCTTTCTTC
Genotype	*Hp1bp3*	TCGTGGTATCGTTATGCGCC<br>GAAAGGTGAGTCTGTCCCCG<br>CCCTTCTGCAACACAGCATC

Table S1. (continued) Garfinkel *et al*

Test	*Hp1bp3*$^{+/+}$ average ± SD			*Hp1bp3*$^{+/-}$ average ± SD			*Hp1bp3*$^{-/-}$ average ± SD		
**Creatinine**	0.18	±	0.04	0.21	±	0.04	0.175	±	0.01
**Ca**	7.89	±	0.72	7.58	±	0.31	7	±	0.06
**Phosphate**	12.25	±	0.78	12.2	±	0.28	12.2	±	0.28
**Glucose**	45	±	4.24	58	±	2.83	46	±	2.83
**Urea**	40.45	±	0.07	40.2	±	4.24	40.4	±	0.00
**Cholesterol**	81.5	±	9.19	76	±	5.66	80	±	0.00
**TP**	2.39	±	0.24	2.38	±	0.08	2.34	±	0.08
**Albumin**	1.045	±	0.01	1.1	±	0.31	0.96	±	0.11
**Globulin**	1.345	±	0.25	1.28	±	0.23	1.38	±	0.03
**Bilirubin**	0.43	±	0.01	0.42	±	0.03	0.58	±	0.14
**Alk Phos**	1279	±	35.36	1272	±	33.94	1116	±	107.48
**SGOT**	246.5	±	47.38	270	±	8.49	300	±	11.31
**SGPT**	64.5	±	27.58	60	±	5.66	60	±	5.66
**Na**	143	±	0.00	144	±	1.41	142.5	±	0.71
**K**	8.9	±	1.13	8	±	0.71	8.35	±	0.92
**Cl**	106.5	±	2.12	106.5	±	0.71	105.5	±	0.71

Table S2: Analysis of serum from newborn mice. Complete litters from heterozygous crosses were collected. Pups were killed and blood was collected immediately, followed by clotting and serum separation. After genotyping, samples were pooled in order to achieve necessary volumes for tests. In all cases, 2 such pools were used for each genotype.
